# NMDA Receptors and NO:cGMP Signaling Pathway Mediate the Diazepam-Induced Sensitization to Withdrawal Signs in Mice

**DOI:** 10.1007/s12640-017-9810-1

**Published:** 2017-09-21

**Authors:** Sylwia Talarek, Joanna Listos, Jolanta Orzelska-Gorka, Anna Serefko, Jolanta Kotlińska

**Affiliations:** 1 Chair and Department of Pharmacology and Pharmacodynamics, Medical University of Lublin, Chodzki 4A, 20-093 Lublin, Poland; 2 Chair and Department of Applied Pharmacy, Medical University of Lublin, Chodźki 1, 20-093 Lublin, Poland

**Keywords:** Diazepam, Sensitization, Withdrawal, NMDA receptor, NO:cGMP pathway

## Abstract

The goal of the present study was to examine the effects of N-methyl—aspartate (NMDA) receptor antagonists—memantine and ketamine and the drugs modifying the NO:cGMP pathway—NG-nitro-L-arginine methyl ester (L-NAME) and 7-nitroindazole (7-NI), the endogenous precursor of NO-L-arginine, and the guanylyl cyclase inhibitor—methylene blue (MB) on the development of sensitization to withdrawal signs precipitated after chronic, interrupted treatment with diazepam, a benzodiazepine receptor agonist, in mice. To develop the sensitization, the mice were divided into groups: continuously and sporadically (with two diazepam-free periods) treated with diazepam (15 mg/kg, sc). To precipitate the withdrawal syndrome (clonic and tonic seizures, and death), pentylenetetrazole (55 mg/kg, sc) with the benzodiazepine receptor antagonist, flumazenil (5.0 mg/kg, ip), were administered after the last injection of diazepam or saline. Memantine (2.5, 5.0 mg/kg), and ketamine (2.5, 5.0 mg/kg), L-NAME (100, 200 mg/kg) and 7-NI (20 and 40 mg/kg), L-arginine (250, 500 mg/kg) and MB (5 and 10 mg/kg) were administered ip in sporadically diazepam-treated mice during the diazepam-free periods. Our results indicated that both NMDA receptor antagonists and drugs that inhibit the NO:cGMP pathway, except L-arginine (the endogenous donor of NO), attenuated the diazepam-induced sensitization to withdrawal signs in mice. Thus, NMDA receptors and the NO:cGMP pathway are involved in the mechanisms of sensitization to benzodiazepine withdrawal.

## Introduction

Benzodiazepines are widely used in the treatment of anxiety disorders and sleep disturbances. Their clinical efficacy is mainly associated with the inhibitory activity of the γ-aminobutyric acid (GABA). Benzodiazepines bind to a specific site on the GABA_A_ receptors that are widely distributed in the postsynaptic neurons and present high affinity to this drug family. Molecular studies demonstrated great diversity in GABA_A_ receptors structure, distribution, and functioning. For example, GABA_A_ receptors that contain α_1_, α_2_, α_3_, or α_5_ subunits are diazepam-sensitive, whereas those that contain α_4_ or α_6_ subunits are diazepam-insensitive. The main disadvantage of the prolonged administration of benzodiazepines is the development of physical dependence and tolerance to their sedative, muscle relaxant and anticonvulsant activity, which limit the clinical relevance in the long-term treatment (Allison and Pratt 2003). Moreover, an abrupt cessation of treatment with benzodiazepines in animal models results in increased levels of anxiety (File 1989), enhanced seizure sensibility (Rundfeldt et al. 1995), tremors, spontaneous convulsions, and body weight loss (Suzuki et al. 1992). The scientists are not united as to the exact mechanism that underlies the development of benzodiazepine dependence, desensitization of GABA/benzodiazepine interaction, and reactions that accompany benzodiazepine withdrawal. Several authors suggest that some modifications at the level of the GABA_A_ receptors and their functioning may partially contribute to the development of benzodiazepine tolerance and dependence. Among these are changes in the composition of GABA_A_ receptors induced by alterations in expression of GABA_A_ receptors, subunit mRNA and subunit protein, reduction in GABA_A_ receptor-mediated fast inhibitory synaptic transmission (Chen et al. 1999), alterations in coupling between benzodiazepine site and GABA receptor-gated chloride channels (Brett and Pratt 1995; Gonsalves and Gallager 1985), or downregulation of benzodiazepine receptor binding in specific brain regions (i.e., cortex, hippocampus, and amygdala). However, the protracted administration of diazepam most probably does not lead to a decrease in GABA_A_ receptor affinity (Fahey et al. 2001). Moreover, it has been postulated that neuroadaptations in other systems should also be taken into consideration.

Glutamatergic neurotransmission and signaling dependent on nitric oxide (NO) make an undeniable contribution to the development of benzodiazepine tolerance and the appearance of the withdrawal symptoms. Both systems play key roles in synaptic plasticity. Furthermore, a significant link between GABAergic, glutamatergic and L-arginine:NO:cGMP pathways has been described (Allison and Pratt 2006; Segovia et al. 1994). Above all, after stimulation of the NMDA receptors-gated ion channel, calcium ions enter the cell and bind to calmodulin. In turn, the Ca^2+^-calmodulin complex enables production of NO from L-arginine under the influence of NOS (Garthwaite and Boulton 1995). Blockage of the NMDA receptor is accompanied by reduced concentration of NO and cGMP (Snyder 1992). It has been suggested that the compensatory mechanisms (i.e., sensitization) in the glutamate signaling may be responsible for the expression of benzodiazepine withdrawal symptoms (Stephens 1995). At first, in response to the enhanced GABAergic activity induced by a chronic administration of benzodiazepines, upregulation of the glutamatergic neurotransmission occurs. After benzodiazepine withdrawal, glutamatergic overactivity is no longer masked by the heightened inhibitory effects of the GABAergic system, and this imbalance may lead to emergence of seizures, increased muscle tone, and anxiety (File and Fernandes 1994). Interestingly, the NMDA receptors seem to be implicated in tolerance to the sedative (File and Fernandes 1994) and anticonvulsant (Koff et al. 1997) effects of benzodiazepines, as well as the onset of withdrawal symptoms, whereas the α-amino-3-hydroxy-5-methylisoxazole-4-propionate (AMPA) receptors seem to be engaged in the withdrawal process only (Steppuhn and Turski 1993). In accordance with the results of Suzuki et al. (1999), the metabotropic glutamate receptors should be involved in the latter process as well, since their antagonists are capable of suppressing the hypersusceptibility to pentylenetetrazole-induced seizure during diazepam withdrawal. Strong evidence also supports the involvement of NO signaling in the mechanisms of drug tolerance and dependence (Babey et al. 1994; Wazlawik and Morato 2002), including the development of tolerance to diazepam-induced motor dysfunction (Talarek et al. 2008). The results of our previous study clearly indicated that the cGMP/NO system may participate in the process of benzodiazepine withdrawal, as the non-selective NOS inhibitors (Nω-Nitro-L-arginine methyl ester and L-NG-nitro arginine) attenuated pentylenetetrazole-induced withdrawal symptoms in mice chronically treated with diazepam (Talarek et al. 2011).

Intermittent administration of diazepam (i.e., 28-day treatment schedule except for the days 5, 10, 15, 20, and 25) may prevent the development of physical dependence as a consequence of the chronic use of this drug (Açıkmeşe et al. 2012). Furthermore, a reduced convulsant threshold in animals subjected to the repeated withdrawal from diazepam treatment has been observed (Ward and Stephens 1998). After cessation of benzodiazepine treatment, mice given diazepam for 3 weeks with 72-h intervals after each week of therapy were more prone to seizures, in comparison to mice who continuously received the drug for 21 days. This phenomenon is widely known as sensitization (Allison and Pratt 2003), and it was also observed for ethanol (Becker et al. 1998). According to the published data (Dunworth and Stephens 1998; Becker et al. 1998), glutamate neurotransmission is partially responsible for this process. Still, upregulation of transmission mediated via AMPA receptors did not seem to be very important in the enhanced seizure activity after diazepam repeated withdrawal, but intensified AMPA receptor binding in brain areas responsible for emotional responses and seizure activity has been seen in mice subjected to 21-day benzodiazepine treatment alone (Allison et al. 2005). On the other hand, upregulation of the NMDA receptor seems to play an important role in the hypersusceptibility of the diazepam-withdrawn mice to seizure incidents (Tsuda et al. 1998b).

The main objective of the present study was to investigate if modulation of either the glutamatergic system or NO-dependent signaling may affect the mechanism of sensitization to diazepam withdrawal signs. We decided to use two different antagonists of the NMDA receptor complex: (i) memantine—an uncompetitive blocker of the NMDA receptor channel and (ii) ketamine—a non-competitive inhibitor of the NMDA receptor complex, and four agents affecting synthesis and/or activity of NO: (i) L-NAME—a non-selective inhibitor of NOS, (ii) 7-nitroindazole—an inhibitor of neural nitric oxide synthase (nNOS), (iii) L-arginine—the endogenous precursor of NO, and (iv) methylene blue (MB)—an inhibitor of nitric oxide-stimulated soluble guanyl cyclase. Activation of guanyl cyclase plays a significant role in one of the NO pathways (Garthwaite and Boulton 1995).

## Experimental Procedures

### Animals

All experiments were carried out on naïve adult male Albino Swiss mice (19–35 g). The animals were housed in the environmentally controlled rooms (12 h light/dark cycle, temperature of 22 ± 1 °C) and were given ad libitum access to water and food. Each experimental group consisted of 8–10 subjects. All procedures were performed in accordance with binding European and Polish legislation acts related to the experimental studies on animal models and were approved by the Local Ethics Committee.

### Drug Administration

The following agents were used in the experiments: diazepam (Relanium, Polfa Warszawa, Poland), N^ω^-Nitro-L-arginine methyl ester hydrochloride (L-NAME, Sigma-Aldrich, USA), 7-nitroindazole (Sigma-Aldrich, USA), L-arginine (Sigma-Aldrich, USA), methylene blue (Sigma-Aldrich, USA), memantine hydrochloride (Lundbeck, Denmark), ketamine hydrochloride (Ketanest, Park Davis, Germany), flumazenil (Hoffman-La Roche, Switzerland), and pentylenetetrazole (Cardiazolum, Polfa, Poland). All of them, except for 7-nitroindazole and flumazenil, were dissolved or diluted in saline. As for flumazenil and 7-nitroindazole, they were suspended in dimethyl sulfoxide (DMSO) and 0.3% Tween 80, respectively, and then diluted in saline. Diazepam, pentylenetetrazole, and saline were given by subcutaneous (sc.) injection, whereas the other agents were administered intraperitoneally (ip). Mice from the control groups received saline. The volume of all administered solutions was 10 ml/kg.

### Procedures

Benzodiazepine dependence in mice was produced by sc administration of diazepam at a daily dose of 15.0 mg/kg for 21 days. In order to show the sensitization to diazepam withdrawal signs, the mice were divided into two groups: (i) the animals were given diazepam for 21 consecutive days (i.e., continuous treatment), (ii) the animals were given diazepam for three 7-day periods that were interspersed with 3-day periods during which they received saline injections (i.e., sporadic treatment). The control animals received saline either as continuous or sporadic treatment. NMDA receptor antagonists—memantine (2.5 or 5.0 mg/kg) and ketamine (2.5 or 5.0 mg/kg) or the agents influencing NO:cGMP pathway—L-NAME (100 or 200 mg/kg), 7-nitroindazole (20 or 40 mg/kg), L-arginine (250 or 500 mg/kg), and methylene blue (5 or 10 mg/kg) were given to mice receiving the sporadic diazepam treatment during the diazepam-free periods (i.e., 3 daily injections in each 3-day period).

The withdrawal signs were observed 48 h after the last injection of diazepam. They were induced by a joint administration of pentylenetetrazole (55.0 mg/kg) and flumazenil (5.0 mg/kg). Immediately after injections, the animals were individually placed in glass cylinders and their behavior was observed for 60 min. Increase in seizure activity (i.e., the number of clonic and tonic convulsions) and death episodes were recorded. The doses and pretreatment schedules were selected on the basis of published studies and the results of our previous experiments (Listos et al. 2008).

### Statistical Analysis

The obtained data were assessed by either the one-way analysis of variance (ANOVA), followed by Tukey-Kramer post hoc test (the number of withdrawal signs), or the Fisher’s exact test (the number of mice developing withdrawal signs). The significance levels were set at 0.05 (*P* < 0.05).

## Results

### The Effects of Co-administration of Flumazenil and Pentylenetetrazole in Mice Subjected to Diazepam Treatment

**Fig. 1:**
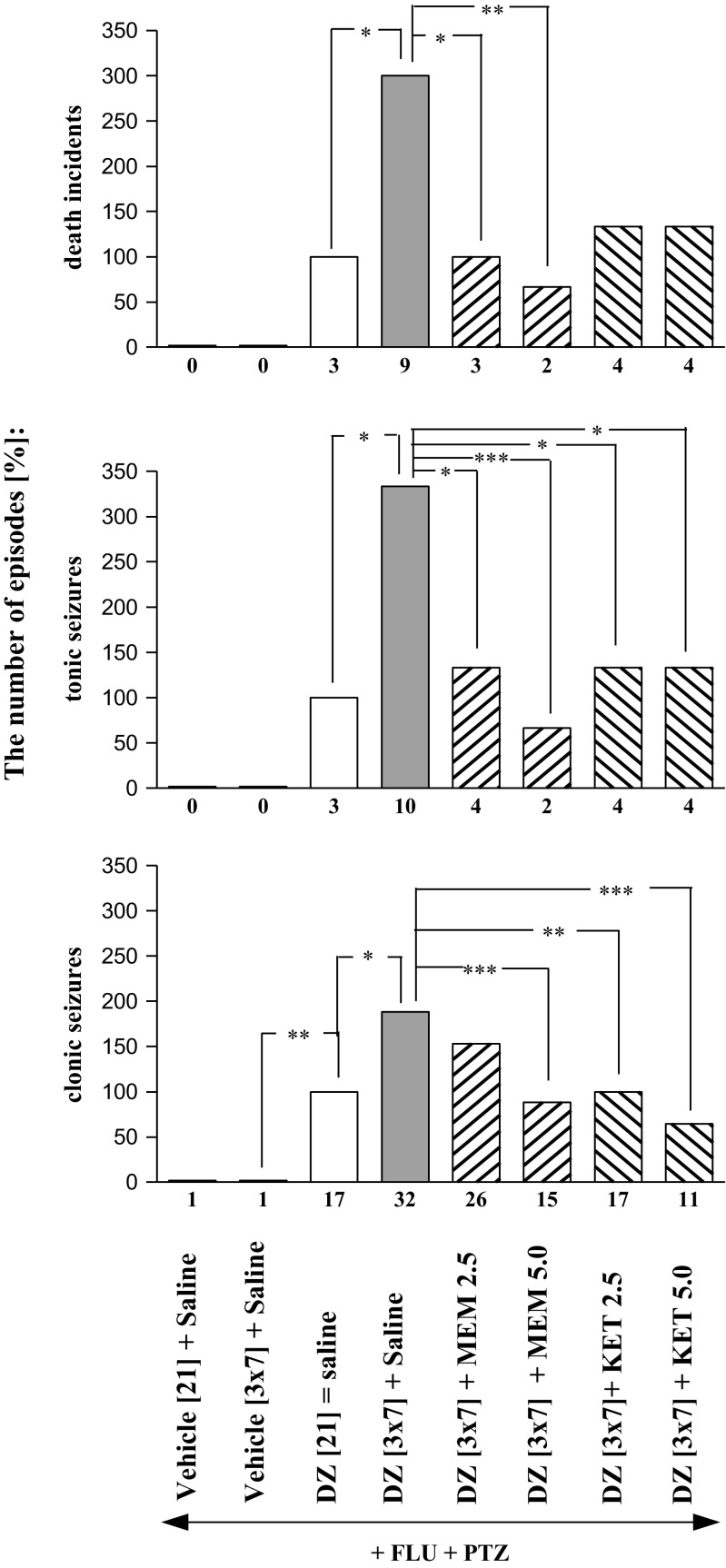
Effects of memantine (MEM; 2.5 and 5.0 mg/kg, *ip*) and ketamine (KET; 2.5; 5.0 mg/kg *ip*) on the development of sensitization to diazepam withdrawal signs. NMDA antagonists were injected in mice once a day for 3 days during diazepam-free periods. The withdrawal signs were induced 48 h after the cessation of diazepam treatment by simultaneous injection of subthreshold dose of pentylenetetrazole (PTZ; 55 mg/kg, *sc*) with flumazenil (FLU; 5.0 mg/kg, *ip*). Data represent the number of clonic, tonic, and death incidents in %. The values of the number of withdrawal signs in mice treated with diazepam (DZ; 21 days) + Saline + FLU + PTZ were assumed to be 100%. The number of clonic and tonic episodes in 10 mice and the mortality rate are presented below X axis. One-way ANOVA: *P < 0.05, **P < 0.01, ***P < 0.001; *N* = 10

**Fig. 2:**
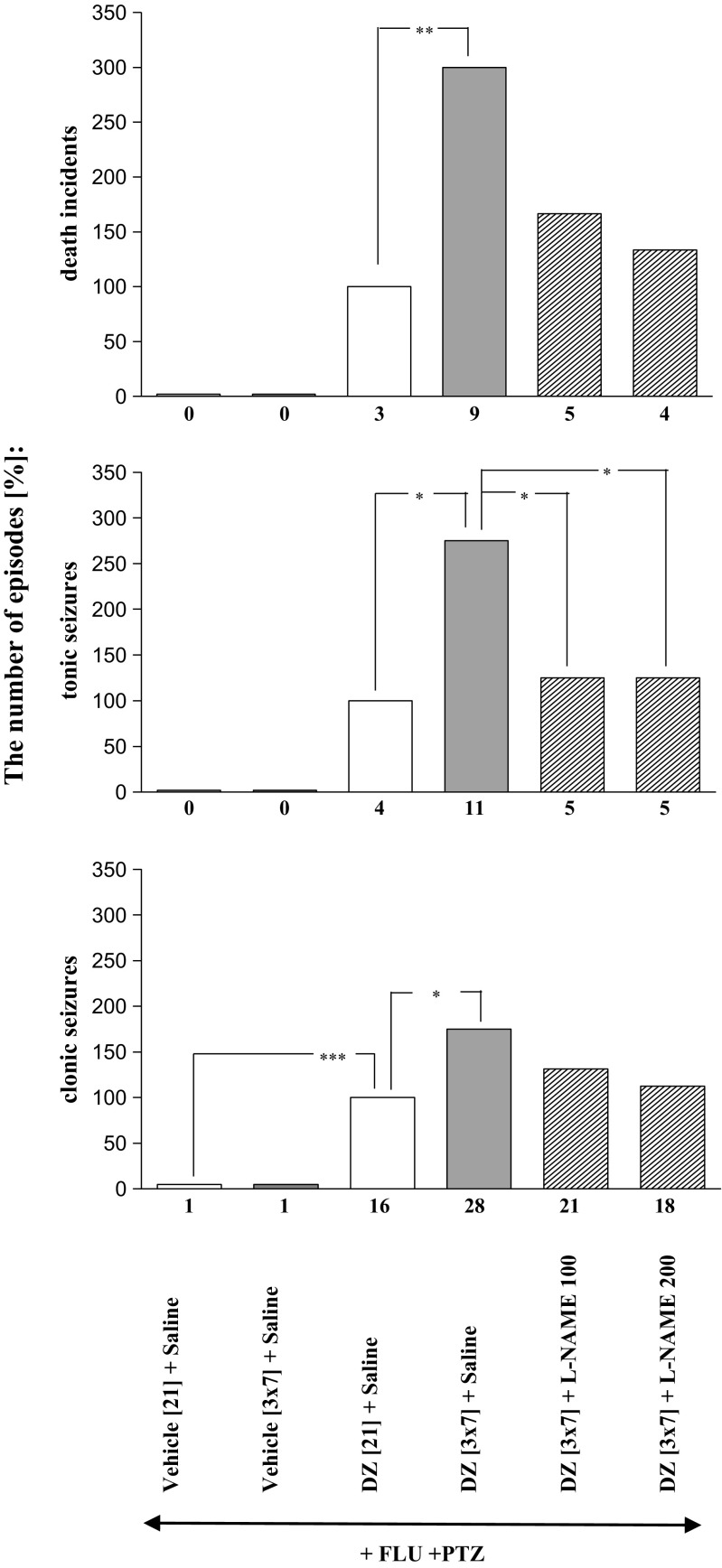
Effects of L-NAME (100 and 200 mg/kg, *ip*) on the development of sensitization to diazepam withdrawal signs. L-NAME was injected in mice once a day for 3 days during diazepam-free periods. The withdrawal signs were induced 48 h after the cessation of diazepam treatment by simultaneous injection of subthreshold dose of pentylenetetrazole (PTZ; 55 mg/kg, *sc*) with flumazenil (FLU; 5.0 mg/kg, *ip*). Data represent the number of clonic, tonic, and death incidents in %. The values of the number of withdrawal signs in mice treated with diazepam (DZ; 21 days) + Saline + FLU + PTZ were assumed to be 100%. The number of clonic and tonic episodes in 10 mice and the mortality rate are presented below X axis. *N* = 10, *P < 0.05, **P < 0.01, ***P < 0.001

**Fig. 3:**
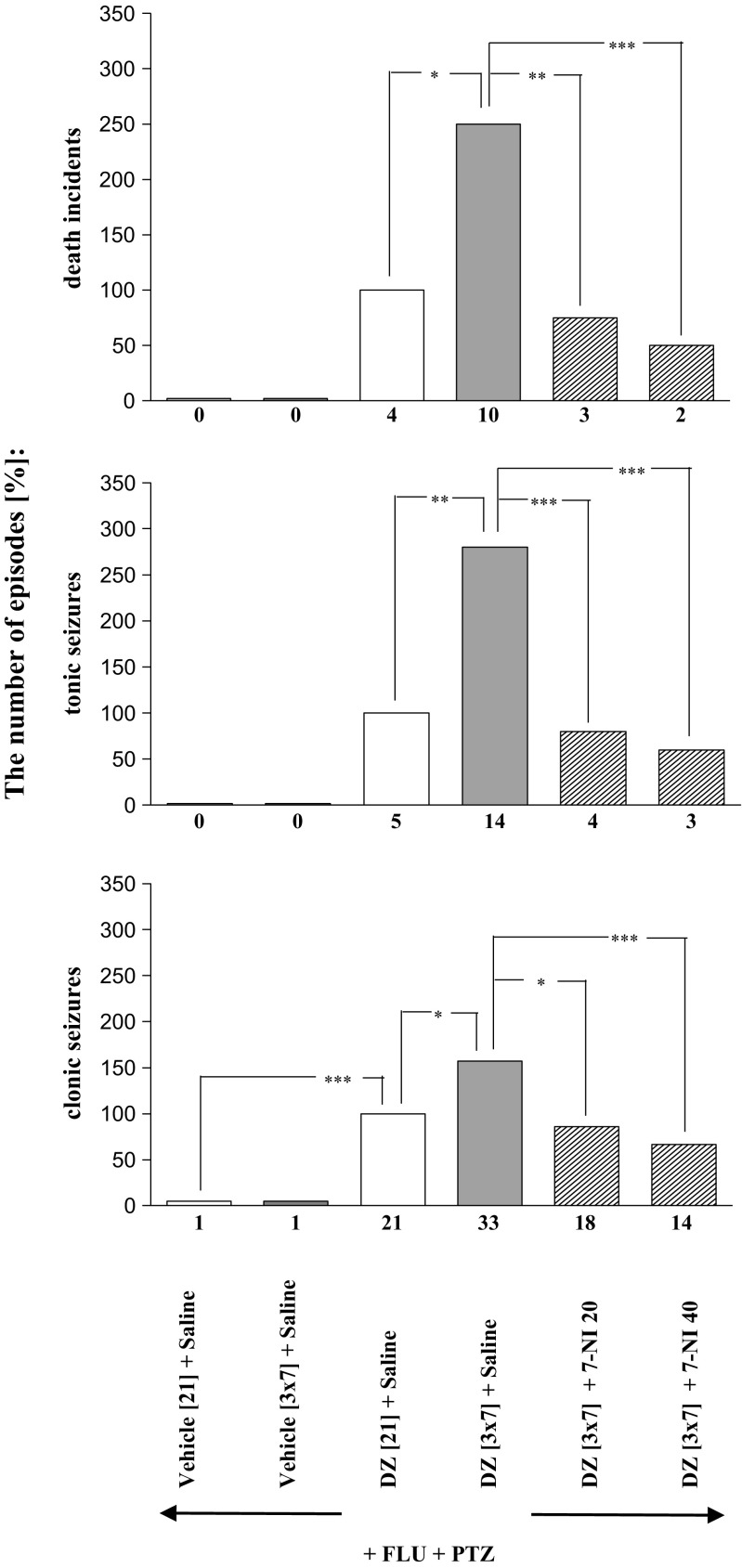
Effects of 7-nitroindazol (7-NI; 20 and 40 mg/kg, *ip*) on the development of sensitization to diazepam withdrawal signs. 7-NI was injected in mice once a day for 3 days during diazepam-free periods. The withdrawal signs were induced 48 h after the cessation of diazepam treatment by simultaneous injection of subthreshold dose of pentylenetetrazole (PTZ; 55 mg/kg, *sc*) with flumazenil (FLU; 5.0 mg/kg, *ip*). Data represent the number of clonic, tonic, and death incidents in %. The values of the number of withdrawal signs in mice treated with diazepam (DZ; 21 days) + Saline + FLU + PTZ were assumed to be 100%. The number of clonic and tonic episodes in 12 mice and the mortality rate are presented below X axis. *N* = 12, *P < 0.05, **P < 0.01, ***P < 0.001

**Fig. 4:**
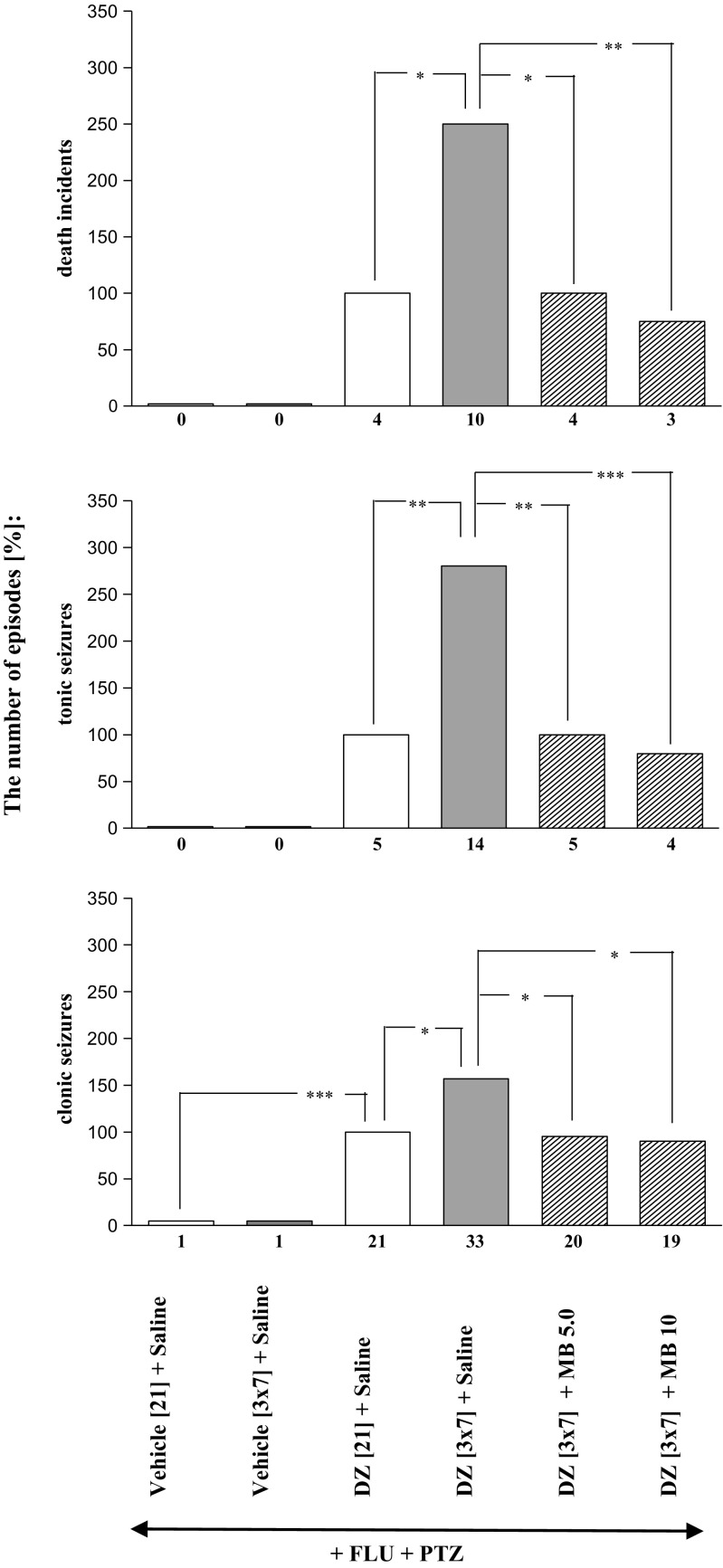
Effects of methylene blue (MB; 5.0 and 10 mg/kg, *ip*) on the development of sensitization to diazepam withdrawal signs. MB was injected in mice once a day for 3 days during diazepam-free periods. The withdrawal signs were induced 48 h after the cessation of diazepam treatment by simultaneous injection of subthreshold dose of pentylenetetrazole (PTZ; 55 mg/kg, *sc*) with flumazenil (FLU; 5.0 mg/kg, *ip*). Data represent the number of clonic, tonic and death incidents in %. The values of the number of withdrawal signs in mice treated with diazepam (DZ; 21 days) + Saline + FLU + PTZ were assumed to be 100%. The number of clonic and tonic episodes in 12 mice and the mortality rate are presented below X axis. *N* = 12, *P < 0.05, **P < 0.01, ***P < 0.001

**Fig. 5:**
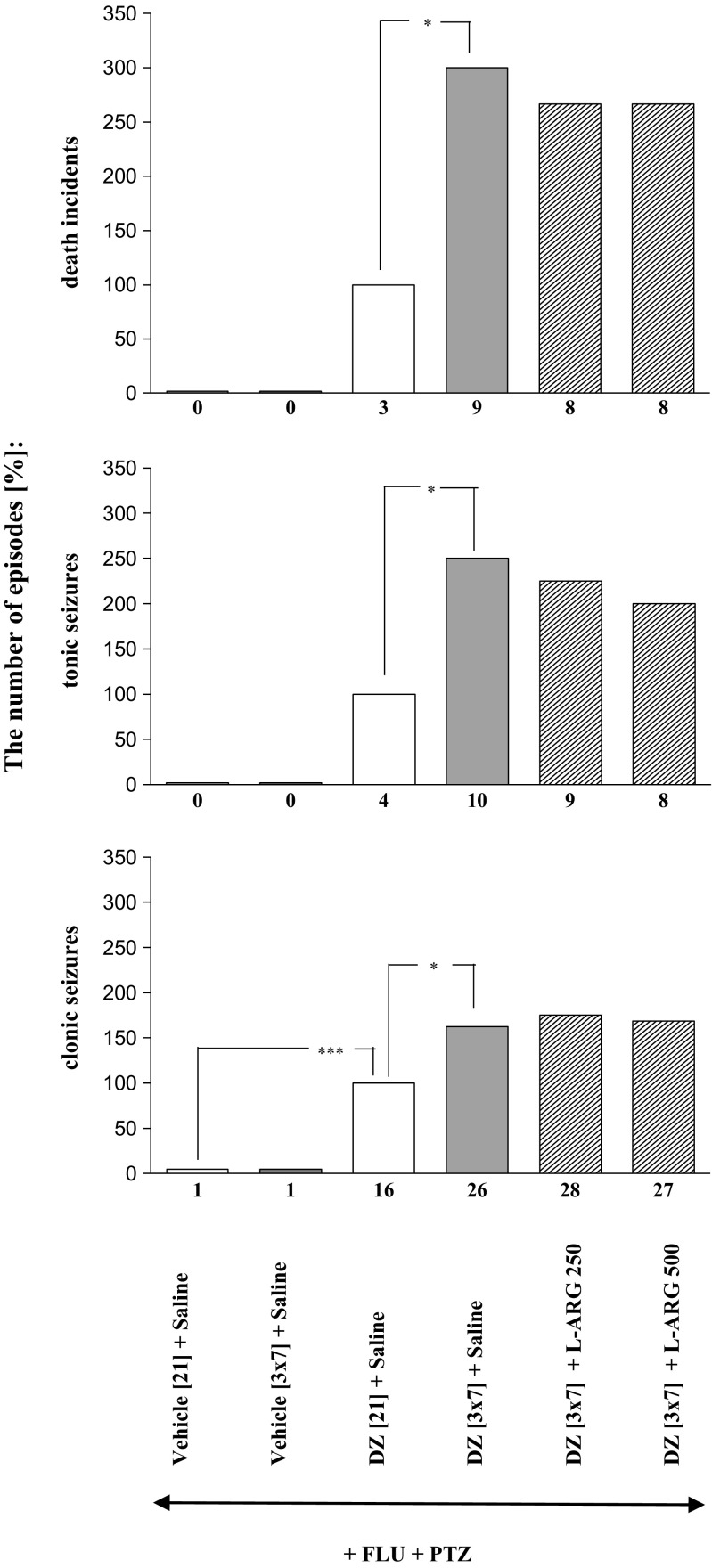
Effects of L-arginine (L-ARG; 250 and 500 mg/kg, *ip*) on the development of sensitization to diazepam withdrawal signs. L-NAME was injected in mice once a day for 3 days during diazepam-free periods. The withdrawal signs were induced 48 h after the cessation of diazepam treatment by simultaneous injection of subthreshold dose of pentylenetetrazole (PTZ; 55 mg/kg, *sc*) with flumazenil (FLU; 5.0 mg/kg, *ip*). Data represent the number of clonic, tonic, and death incidents in %. The values of the number of withdrawal signs in mice treated with diazepam (DZ; 21 days) + Saline + FLU + PTZ were assumed to be 100%. The number of clonic and tonic episodes in 10 mice and the mortality rate are presented below X axis. *N*=10. *P<0.05, ***P<0.001

Concurrent administration of flumazenil (5.0 mg/kg, ip) and pentylenetetrazole (55.0 mg/kg sc) induced withdrawal signs (clonic seizures, tonic convulsions and death episodes) in animals chronically treated with diazepam. However, this effect was more pronounced in the case of animals receiving the sporadic treatment. Both number of convulsions and death episodes were significantly higher in mice treated in the scheme with diazepam-free periods, as compared to the control group given the benzodiazepine for 21 straight consecutive days (Figs. 1, 2, 3, 4, and 5). As shown in Table 1, the seizure threshold was observed after administration of pentylenetetrazole in saline-treated mice. There were no significant changes in seizure activity after injection of pentylenetetrazole in mice both continuously and sporadically treated with diazepam. The seizure activity was not also changed after co-administration of pentylenetetrazole with flumazenil in saline-treated mice. The number of mice developing tonic seizures and death incidents was statistically bigger in the group receiving diazepam sporadically, in comparison to that which received diazepam continuously (*P* < 0.05).

**Table 1:** Effects of subthreshold dose of pentylenetetrazole (PTZ; 55.0 mg/kg, *sc*) alone, and PTZ with flumazenil (FLU; 5.0 mg/kg, *ip*) on chronically diazepam (DZ; 15 mk/kg, *sc*) treated mice. To develop the sensitization to DZ withdrawal signs, the mice were divided into two groups: continuously treated with DZ [21] and intermittently treated with DZ [3 × 7] with two drug-free periods (3 days). PTZ and FLU were injected 48 h after the last DZ injection. Data represent the number of mice responding with withdrawal signs. *N* = 10;12. ^▲^ *P* < 0.05, ^▲▲▲^ *P* < 0.001 vs vehicle + FLU + PTZ, *P < 0.05 vs DZ [21] + FLU + PTZ (Fisher’s exact test)

Substance [mg/kg]	The number of mice developing:		
	Clonic seizures	Tonic seizures	Death incidents
Vehicle [21] + PTZ	1/10	0/10	0/10
	1/12	0/12	0/12
Vehicle [3 × 7] + PTZ	1/10	0/10	0/10
	1/12	0/12	0/12
DZ [21] + PTZ	2/10	1/10	0/10
	3/12	1/12	0/12
DZ [3 × 7] + PTZ	3/10	1/10	0/10
	3/12	2/12	0/12
Vehicle [21] + FLU + PTZ	1/10	0/10	0/10
	1/12	0/10	0/12
Vehicle [3 × 7] + FLU + PTZ	1/10	0/10	0/10
	1/12	0/12	0/12
DZ [21] + FLU + PTZ	9/10^▲▲▲^	4/10	3/10
	11/12^▲▲▲^	5/12^▲^	4/12^▲^
DZ [3 × 7] + FLU + PTZ	10/10^▲▲▲^	10/10^▲▲▲,^*	9/10^▲▲▲,^*
	12/12^▲▲▲^	11/12^▲▲▲,^*	10/12^▲▲▲,^*

### The Effects of the NMDA Receptor Antagonists on the Development of Diazepam Withdrawal-Induced Sensitization

As illustrated in Fig. 1, one-way ANOVA indicated that administration of NMDA antagonists, memantine and ketamine, in the diazepam-free periods elicited a statistically significant effect in the pentylenetrazole-induced seizures in sporadically diazepam-treated mice (clonic seizures—*F*(5,59) = 6.807, tonic seizures—*F*(5,59) = 7.360, and death incidents—*F*(5,59) = 18.74 for memantine, and *F*(5,59) = 5.064, tonic seizures—*F*(5,59) = 6.688 and death incidents—*F*(5,59) = 17.40 for ketamine; *P* < 0.0001) as compared to the group receiving injections of saline in the diazepam-free periods.

The Tukey post hoc analysis showed that memantine at the dose of 2.5 mg/kg significantly diminished the number of tonic convulsions (*P* < 0.05) and death incidents (*P* < 0.05), whereas the higher dose of memantine (5.0 mg/kg) decreased the number of clonic seizures (*P* < 0.001), tonic seizures (*P* < 0.001) and death episodes (*P* < 0.01). In the analogous studies on ketamine, both applied doses of the NMDA receptor antagonist (i.e., 2.5 and 5.0 mg/kg) reduced the number of diazepam withdrawal clonic convulsions (*P* < 0.01, *P* < 0.001, respectively) and tonic convulsions (*P* < 0.01 for both doses of memantine), but they did not affect the incidence of death.

The Fisher exact test revealed that administration of memantine at the dose of 2.5 and 5.0 mg/kg significantly reduced the number of mice developing tonic convulsions (*P* < 0.01 and *P* < 0.001, respectively) and death (*P* < 0.05 and *P* < 0.01, respectively). The higher dose of memantine (5.0 mg/kg) also decreased the number of mice reacting with clonic seizures (*P* < 0.05). In the analogous studies, ketamine at the doses of 2.5 and 5.0 mg/kg reduced the number of mice developing diazepam withdrawal tonic convulsions (*P* < 0.05) and mortality (*P* < 0.05), but they did not affect the number of mice developing clonic seizures.

### The Effects of the Agents Influencing L-Arginine-NO-cGMP Signaling Pathway on the Development of Diazepam Withdrawal-Induced Sensitization

As shown in Figs. 2, 3, 4, and 5 and Table 1, one-way ANOVA revealed that the pentylenetetrazole-induced seizure activity and mortality were significantly increased after withdrawal from chronic, intermittent treatment with diazepam (*P* < 0.001).

The Tukey post hoc test revealed that administration of L-NAME at the doses of 100 and 200 mg/kg during the diazepam-free periods significantly decreased the number of tonic convulsions (*P* < 0.05), but it did not affect the number of clonic seizures or death episodes (Fig. 3). Moreover, pretreatment with 7-nitroindazole at the doses of 20 and 40 mg/kg during the diazepam-free periods significantly reduced the number of withdrawal clonic seizures (*P* < 0.05 and *P* < 0,001, respectively), tonic seizures (*P* < 0.001) and death incidents (*P* < 0.01 and *P* < 0.001, respectively) analyzed in the study (Fig. 3). As demonstrated in Fig. 4, administration of methylene blue at the doses of 5.0 and 10.0 mg/kg during the diazepam-free periods also significantly reduced the number of clonic convulsions (*P* < 0.05), tonic convulsions (*P* < 0.01 and *P* < 0.001, respectively) and death incidents (*P* < 0.05 and *P* < 0.01, respectively). However, neither withdrawal seizure activity nor death incidents were affected by administration of L-arginine (250 and 500 mg/kg) during two diazepam-free periods in sporadically diazepam-treated mice (Fig. 5).

As shown in Table 2, the Fisher exact test reveals that administration of L-NAME at the doses of 100 and 200 mg/kg during the diazepam-free periods significantly decreased the number of mice developing tonic convulsions (*P* < 0.05) and mortality (*P* < 0.05). What is more, at a higher dose (200 mg/kg), L-NAME decreased the number of mice developing clonic seizures (*P* < 0.05)). Pretreatment with 7-nitroindazole at the doses of 20 and 40 mg/kg during the diazepam-free periods also significantly reduced the number of mice developing tonic seizures (*P* < 0.01) and mortality (*P* < 0.01 and *P* < 0.001, respectively). Moreover, administration of methylene blue at the doses of 5.0 and 10.0 mg/kg during the diazepam-free periods significantly reduced the number of mice reacting with tonic convulsions (*P* < 0.05 and *P* < 0.01 respectively) and death (*P* < 0.05). However, the number of mice developing withdrawal seizure activity and death were not affected by administration of L-arginine (250 and 500 mg/kg) during two diazepam-free periods in sporadically diazepam-treated mice.

**Table 2:** Effect of L-arginine:NO:cGMP modulators and NMDA antagonists on the development of sensitization to diazepam withdrawal signs. NMDA receptor antagonists—memantine (2.5 or 5.0 mg/kg, *ip*) and ketamine (2.5 or 5.0 mg/kg, *ip*) or the drugs modulating NO:cGMP pathway—L-NAME (100 or 200 mg/kg, *ip*), 7-nitroindazole (7-NI, 20 or 40 mg/kg, *ip*), L-arginine (L-ARG, 250 or 500 mg/kg, *ip*), and methylene blue (MB, 5 or 10 mg/kg, *ip*) were given to mice receiving the sporadic diazepam treatment during the diazepam-free periods (i.e., 3 daily injections in each 3-day period). Data represent the number of mice responding with withdrawal signs. *N* = 10;12. *P < 0.05, **P < 0.01, ***P < 0.001 vs DZ [3 × 7] + Saline +FLU + PTZ (Fisher’s exact test)

Substance [mg/kg]	The number of mice developing:		
	Clonic seizures	Tonic seizures	Death incidents
DZ [3 × 7] + Saline + FLU + PTZ	10/10	10/10	9/10
	12/12	11/10	10/12
DZ [3 × 7] + MEM 2.5 + FLU + PTZ	10/10**	4/10**	4/10*
DZ [3 × 7] + MEM 5.0 + FLU + PTZ	6/10*	2/10***	2/10**
DZ [3 × 7] + KET 2.5 + FLU + PTZ	9/10	5/10*	4/10*
DZ [3 × 7] + KET 5.0 + FLU + PTZ	8/10	5/10*	4/10*
DZ [3 × 7] + L-NAME 100 + FLU + PTZ	9/10	5/10*	5/10*
DZ[3 × 7] + L-NAME 200 + FLU + PTZ	6/10*	5/10*	4/10*
DZ [3 × 7] + 7-NI 20 + FLU + PTZ	9/12	4/12**	3/12*
DZ[3 × 7] + 7-NI 40 + FLU + PTZ	9/12	3/12**	2/12**
DZ [3 × 7] + MB 5.0 + FLU + PTZ	11/12	5/12*	4/12*
DZ [3 × 7] + MB 10 + FLU + PTZ	10/12	4/12**	3/12*
DZ[3 × 7] + L-ARG 250 + FLU + PTZ	10/10	9/10	8/10
DZ [3 × 7] + L-ARG 500 + FLU + PTZ	9/10	8/10	8/10

## Discussion

It is widely known that discontinuation of the prolonged diazepam treatment in rodents results in a significant decrease in the seizure threshold for convulsion. However, it has been demonstrated that mice which have experienced a single or repeated withdrawal from diazepam treatment present different patterns of anxious behavior and sensitivity to seizures. Repeated withdrawal from benzodiazepine treatment is associated with increased seizure sensitivity and decreased withdrawal-induced anxiety and aversion (Ward and Stephens 1998). Furthermore, it was suggested the intensification of diazepam withdrawal signs after sporadic benzodiazepines treatment in comparison with continuously benzodiazepine treated mice (Listos et al. 2006).

As predicted, in the present study, animals given diazepam sporadic treatment (i.e., 15.0 mg/kg/day for three 7-day periods, each interspersed with 3 diazepam-free days) responded more profoundly to the proconvulsant effects of flumazenil (an antagonist of the benzodiazepin receptor) co-administered with a sub-effective dose of pentylenetetrazole than did their counterparts from the group receiving the continuous treatment (i.e., 15 mg/kg/day for 21 days). This effect is defined as the sensitization of withdrawal signs and its mechanisms are poorly described in the literature (Listos et al. 2006; Ward and Stephens 1998).

It is known that both glutamate and NO signaling play an important role in the development of drug dependence, since blockage of the NMDA receptor complex or administration of NOS inhibitors attenuated opioid (Gabra et al. 2005; Trujillo and Akil 1991; Vaupel et al. 1997), ethanol (Liljequist 1991; Uzbay et al. 1997), and nicotine (Jain et al. 2008a, b) withdrawal signs in animal models. Previously, we demonstrated that the NMDA receptor antagonists (memantine and ketamine), as well as NOS inhibitors: (N^G^-nitro-L-arginine, L-NAME, and 7-nitroindazole) prevented the development of tolerance and/or expression of tolerance to motor impairing effect of diazepam (Talarek et al. 2008, 2016), whereas L-arginine facilitated the development of diazepam-induced tolerance to motor impairment (Talarek et al. 2008).

The major findings of our present experiments is that the inhibition of the NMDA receptor complex with memantine and ketamine suppressed the development of diazepam withdrawal sensitization, though the latter compound did not affect the incidence of death. Among the agents influencing L-arginine-NO-cGMP pathway, only the precursor of NO (i.e., L-arginine) did not influence the process of sensitization to seizures in animals repeatedly withdrawn from diazepam, whereas administration of either nNOS or NO-stimulated soluble guanyl cyclase exerted the most potent inhibitory effect. Moreover, 7-NI and MB significantly decreased all measured parameters (clonic seizures, tonic convulsions, and deaths). It could be possible that the tested doses of L-arginine (i.e., 250 and 500 mg/kg) were not high enough to produce a significant effect, since this agent is also used in other physiological pathways, independent of NO synthesis (Raasch et al. 2001). Though there are three isoforms of NOS that can be found in the CNS (endothelial, inducible, and neuronal), nNOS seems to be most important for the involvement of NO-mediated signaling in drug tolerance and dependence (Vaupel et al. 1997). Petros et al. (1991) found out that non-selective inhibitors of NOS may induce vasoconstriction and increase blood pressure that, in turn, is one of the symptoms of drug withdrawal. Therefore, in our experiments, along with application of the non-selective inhibitor of NOS—L-NAME, we also decided to use 7-NI—a selective inhibitor of nNOS that is deprived of the vasopressor activity (Moore et al. 1993).

Though the reports on the effects of NMDA and NO signaling on the development of diazepam withdrawal-induced sensitization are scarce, our results are in a general compliance with the available literature data concerning benzodiazepine withdrawal signs. However, direct comparison of these studies is difficult because of the differences in treatment paradigm used such us dose, period and model of treatment. The study of Dunworth and Stephens (1998) demonstrated that sensitization to the convulsive effects of pentylenetetrazole in mice repeatedly withdrawn from diazepam treatment was prevented by administration of CGP 39551—an antagonist of the NMDA receptor. In this experiment, NMDA receptor ligand was given ip at a dose of 20 mg/kg during the 3-day breaks in diazepam treatment, as it was performed in our study. Furthermore, other studies showed that 14-day infusion of 3-[(+)-2-carboxypiperazin-4-yl]-propyl-1-phosphonate (CPP) during the “active phase” of diazepam withdrawal (i.e., between the 4th and 21st day after treatment cessation) eliminated the signs of benzodiazepine dependence (Steppuhn and Turski 1993), while pretreatment with MK-801 (50 μg/kg, sc) or ifenprodil (20 mg/kg, ip) effectively inhibited pentylenetetrazole-induced and/or methyl-6,7-dimethoxy-4-ethyl-β-carboline-3carboxylate(DMCM)-induced seizures in diazepam-withdrawn mice (Tsuda et al. 1997b, 1998b). Interestingly, the suppression pattern of diazepam withdrawal signs may be different depending on the applied NMDA receptor ligand—e.g. MK-801 (a non-competitive antagonist of the NMDA receptor complex acting at the ion channel sites) suppressed autonomic, emotional and motor withdrawal signs, as well as body loss; whereas ifenprodil was effective in amelioration of the motor and emotional signs (Tsuda et al. 1998d). In a rat model, administration of another NMDA antagonist—2-amino-7-phosphonoheptanoate, into the dorsal periaqueductal gray, and, in consequence, blocking the glutamatergic transmission, alleviated the effects of the diazepam withdrawal (Souza-Pinto et al. 2007). Similarly, to the pattern observed in our present work, mice chronically given diazepam presented attenuated pentylenetetrazole-induced withdrawal symptoms after treatment with L-NAME or N^G^-nitro-L-arginine (Talarek et al. 2011), while L-arginine did not suppress diazepam withdrawal-induced hyperexcitability in the pentylenetetrazole model (Tsuda et al. 1998c). However, L-arginine did exert an inhibitory effect in the electroshock model (Nidhi et al. 2000).

The pharmacological mechanisms of benzodiazepine dependence and withdrawal are complex and still not fully understood. The early studies provided evidence that chronic treatment of rats with diazepam caused a loss in the ability of benzodiazepines to potentiate GABA-stimulated chloride influx (Marley and Gallager 1989) or GABA to potentiate benzodiazepine radioligant binding (Gallager et al. 1984) in cortex. Furthermore, it was shown that interruption of prolonged treatment with diazepam in rats induced the molecular changes in the GABA_A_ receptor, such as the decrease in the density and expression of subunits α1 and α2 in the neurons of the cereral cortex and hippocampus (Ramerstorfer et al. 2011) These pre- and postsynaptic changes, such as a decrease in GABA synthesis and/or release, and the decrease in the expression and composition of GABA_A_ receptors could underlie the observed withdrawal phenomena following termination of the diazepam treatment (Allison and Pratt 2006; Calixto 2016). There are also reports indicating that chronic activation of the GABAergic system during benzodiazepine treatment may perturbate glutamatergic transmission, an opposing to GABA neurotransmitter system in brain (Allison and Pratt 2003; Bateson 2002). It was suggested that the hypersusceptibility to convulsive effects after diazepam withdrawal may be a consequence of the alterations in the NO production via NO synthase and/or the overactivity of the NMDA receptor function (Tsuda et al. 1997a, 1998d), since the anatomical and functional relationship between NMDA/NO/cGMP pathway and the inhibitory transmission dependent on the GABA_A_ was described (Fedele et al. 2007). Besides, NO and glutamate may potentiate mutual release or production (Lin et al. 2000). Overexpression of mRNA for the NR1 and NR2B NMDA receptor subunits was observed in the hippocampus of rats tolerant to diazepam (Perez et al. 2003). This increase in mRNA expression along with the associated development of tolerance to hypolocomotive effect of diazepam were successfully prevented by the pretreatment with MK-801 (Almiron et al. 2004). Furthermore, upregulation of NR1 and NR2B (but not NR2A) subunit proteins of the NMDA receptor complex was detected in the cerebrocortical tissue of diazepam-withdrawn rats (Tsuda et al. 1998a). The findings would be in compliance with the reports concerning effectivity of ifenprodil (i.e., a negative modulator of the NMDA receptor complex selectively binding to the NR1/NR2B receptor subtype) in inhibition of the experimentally induced seizures in diazepam-withdrawn animals (Tsuda et al. 1998b). Accordingly, the modifications after removal of GABAergic inhibition concern not only the NMDA receptors, but also the AMPA receptors, and their involvement depends on the phase of benzodiazepine withdrawal process (Crepel et al. 1997). Herein, the AMPA receptors seem to be crucial in initiation of the withdrawal symptoms, whereas the NMDA receptors play an important role during the later stage (Steppuhn and Turski 1993). In fact, alterations in AMPA receptor binding or the levels of mRNA of the GluR1 or GluR2 AMPA receptor subunits following withdrawal of diazepam were discovered in brain areas implicated in anxiety (i.e., hippocampus, amygdala, thalamus, or limbic-associated cortex) (Allison and Pratt 2006). The results of our present study partially confirm the data that the removal of GABAergic inhibition is accompanied by alteration of both glutamatergic and NO-dependent neurotransmissions. However, further experiments are needed to explain the precise mechanisms underlying the process of sensitization to withdrawal syndrome after chronic, intermittent administration of benzodiazepines.

Summarizing, our studies provide some new data on the development of diazepam-induced withdrawal sensitization in mice. Two main findings should be particularly underlined: (1) chronic, interrupted treatment with diazepam results in the intensified seizure sensitivity; (2) the NMDA receptors and nitric oxide pathway are involved in the development of diazepam-induced withdrawal sensitization in mice.

## References

[CR1] Acikmese B, Haznedar S, Hatipoglu I, Enginar N (2012). Evaluation of anxiolytic effect and withdrawal anxiety in chronic intermittent diazepam treatment in rats. Behav Pharmacol.

[CR2] Allison C, Pratt JA (2003). Neuroadaptive processes in GABAergic and glutamatergic systems in benzodiazepine dependence. Pharmacol Ther.

[CR3] Allison C, Pratt JA (2006). Differential effects of two chronic diazepam treatment regimens on withdrawal anxiety and AMPA receptor characteristics. Neuropsychopharmacology.

[CR4] Allison C, Pratt JA, Ripley TL, Stephens DN (2005). Alpha-Amino-3-hydroxy-5-methylisoxazole-4-propionate receptor autoradiography in mouse brain after single and repeated withdrawal from diazepam. Eur J Neurosci.

[CR5] Almiron RS, Perez MF, Ramirez OA (2004). MK-801 prevents the increased NMDA-NR1 and NR2B subunits mRNA expression observed in the hippocampus of rats tolerant to diazepam. Brain Res.

[CR6] Babey AM, Kolesnikov Y, Cheng J, Inturrisi CE, Trifilletti RR, Pasternak GW (1994). Nitric oxide and opioid tolerance. Neuropharmacology.

[CR7] Bateson AN (2002). Basic pharmacologic mechanisms involved in benzodiazepine tolerance and withdrawal. Curr Pharm Des.

[CR8] Becker HC, Veatch LM, Diaz-Granados JL (1998). Repeated ethanol withdrawal experience selectively alters sensitivity to different chemoconvulsant drugs in mice. Psychopharmacology.

[CR9] Brett RR, Pratt JA (1995). Changes in benzodiazepine-GABA receptor coupling in an accumbens-habenula circuit after chronic diazepam treatment. Br J Pharmacol.

[CR10] Calixto E (2016). GABA withdrawal syndrome: GABAA receptor, synapse, neurobiological implications and analogies with other abstinences. Neuroscience.

[CR11] Chen S, Huang X, Zeng XJ, Sieghart W, Tietz EI (1999). Benzodiazepine-mediated regulation of alpha1, alpha2, beta1-3 and gamma2 GABA(A) receptor subunit proteins in the rat brain hippocampus and cortex. Neuroscience.

[CR12] Crepel V, Khazipov R, Ben-Ari Y (1997). Blocking GABA(A) inhibition reveals AMPA- and NMDA-receptor-mediated polysynaptic responses in the CA1 region of the rat hippocampus. J Neurophysiol.

[CR13] Dunworth SJ, Stephens DN (1998). Sensitisation to repeated withdrawal, in mice treated chronically with diazepam, is blocked by an NMDA receptor antagonist. Psychopharmacology.

[CR14] Fahey JM, Pritchard GA, Grassi JM, Pratt JS, Shader RI, Greenblatt DJ (2001). Pharmacodynamic and receptor binding changes during chronic lorazepam administration. Pharmacol Biochem Behav.

[CR15] Fedele E, Ansaldo MA, Varnier G, Raiteri M (2007). Benzodiazepine-sensitive GABAA receptors limit the activity of the NMDA/NO/cyclic GMP pathway. JNC.

[CR16] File SE (1989). Chronic diazepam treatment: effect of dose on development of tolerance and incidence of withdrawal in an animal test of anxiety. Hum Psychopharmacol.

[CR17] File SE, Fernandes C (1994). Dizocilpine prevents the development of tolerance to the sedative effects of diazepam in rats. Pharmacol Biochem Behav.

[CR18] Gabra BH, Afify EA, Daabees TT, Abou Zeit-Har MS (2005). The role of the NO/NMDA pathways in the development of morphine withdrawal induced by naloxone in vitro. Pharmacol Res.

[CR19] Gallager DW, Lakoski JM, Gonsalves SF, Rauch SL (1984). Chronic benzodiazepine treatment decreases postsynaptic GABA sensitivity. Nature.

[CR20] Garthwaite J, Boulton CL (1995). Nitric oxide signaling in the central nervous system. Annu Rev Physiol.

[CR21] Gonsalves SF, Gallager DW (1985). Spontaneous and RO 15-1788-induced reversal of subsensitivity to GABA following chronic benzodiazepines. Eur J Pharmacol.

[CR22] Jain R, Mukherjee K, Balhara YP (2008). The role of NMDA receptor antagonists in nicotine tolerance, sensitization, and physical dependence: a preclinical review. Yonsei Med J.

[CR23] Jain R, Mukherjee K, Mohan D (2008). Effects of nitric oxide synthase inhibitors in attenuating nicotine withdrawal in rats. Pharmacol Biochem Behav.

[CR24] Koff JM, Pritchard GA, Greenblatt DJ, Miller LG (1997). The NMDA receptor competitive antagonist CPP modulates benzodiazepine tolerance and discontinuation. Pharmacology.

[CR25] Liljequist S (1991). The competitive NMDA receptor antagonist, CGP 39551, inhibits ethanol withdrawal seizures. Eur J Pharmacol.

[CR26] Lin HC, Kang BH, Wan FJ, Huang ST, Tseng CJ (2000). Reciprocal regulation of nitric oxide and glutamate in the nucleus tractus solitarii of rats. Eur J Pharmacol.

[CR27] Listos J, Malec D, Fidecka S (2006). Adenosine receptor antagonists intensify the benzodiazepine withdrawal signs in mice. Pharmacol Rep.

[CR28] Listos J, Talarek S, Fidecka S (2008) Adenosine receptor agonists attenuate the development of diazepam withdrawal-induced sensitization in mice. Eur J Pharmacol 588(1):72–77 10.1016/j.ejphar.2008.04.011 18466897

[CR29] Marley RJ, Gallager DW (1989). Chronic diazepam treatment produces regionally specific changes in GABA-stimulated chloride influx. Eur J Pharmacol.

[CR30] Moore PK, Wallace P, Gaffen Z, Hart SL, Babbedge RC (1993). Characterization of the novel nitric oxide synthase inhibitor 7-nitro indazole and related indazoles: antinociceptive and cardiovascular effects. Br J Pharmacol.

[CR31] Nidhi G, Bhargava VK, Pandhi P (2000). Tolerance to and withdrawal from anticonvulsant action of diazepam: role of nitric oxide. Epilepsy Behav.

[CR32] Perez MF, Salmiron R, Ramirez OA (2003). NMDA-NR1 and -NR2B subunits mRNA expression in the hippocampus of rats tolerant to diazepam. Behav Brain Res.

[CR33] Petros A, Bennett D, Vallance P (1991). Effect of nitric oxide synthase inhibitors on hypotension in patients with septic shock. Lancet.

[CR34] Raasch W, Schäfer U, Chun J, Dominiak P (2001) Biological significance of agmatine, an endogenous ligand at imidazoline binding sites. Br J Pharmacol 133:755–780 10.1038/sj.bjp.0704153 PMC1572857 11454649

[CR35] Ramerstorfer J, Furtmüller R, Sarto-Jackson I, Varagic Z, Sieghart W, Ernst M (2011). The GABAA receptor alpha+beta- interface: a novel target for subtype selective drugs. J Neurosci.

[CR36] Rundfeldt C, Wlaz P, Honack D, Loscher W (1995). Anticonvulsant tolerance and withdrawal characteristics of benzodiazepine receptor ligands in different seizure models in mice. Comparison of diazepam, bretazenil and abecarnil. J Pharmacol Exp Ther.

[CR37] Segovia G, Porras A, Mora F (1994). Effects of a nitric oxide donor on glutamate and GABA release in striatum and hippocampus of the conscious rat. Neuroreport.

[CR38] Snyder SH (1992). Nitric oxide: first in a new class of neurotransmitters?. Science.

[CR39] Souza-Pinto LF, Castilho VM, Brandao ML, Nobre MJ (2007). The blockade of AMPA-kainate and NMDA receptors in the dorsal periaqueductal gray reduces the effects of diazepam withdrawal in rats. Pharmacol Biochem Behav.

[CR40] Stephens DN (1995). A glutamatergic hypothesis of drug dependence: extrapolations from benzodiazepine receptor ligands. Behav Pharmacol.

[CR41] Steppuhn KG, Turski L (1993). Diazepam dependence prevented by glutamate antagonists. Proc Natl Acad Sci U S A.

[CR42] Suzuki T, Lu MS, Motegi H, Yoshii T, Misawa M (1992). Genetic differences in the development of physical dependence upon diazepam in Lewis and Fischer 344 inbred rat strains. Pharmacol Biochem Behav.

[CR43] Suzuki T, Shimizu N, Tsuda M, Soma M, Misawa M (1999). Role of metabotropic glutamate receptors in the hypersusceptibility to pentylenetetrazole-induced seizure during diazepam withdrawal. Eur J Pharmacol.

[CR44] Talarek S, Listos J, Fidecka S (2008). Role of nitric oxide in the development of tolerance to diazepam-induced motor impairment in mice. Pharmacol Rep.

[CR45] Talarek S, Listos J, Fidecka S (2011). Effect of nitric oxide synthase inhibitors on benzodiazepine withdrawal in mice and rats. Pharmacol Rep.

[CR46] Talarek S, Orzelska-Gorka J, Listos J, Serefko A, Poleszak E, Fidecka S (2016). Effects of NMDA antagonists on the development and expression of tolerance to diazepam-induced motor impairment in mice. Pharmacol Biochem Behav.

[CR47] Trujillo KA, Akil H (1991). Inhibition of morphine tolerance and dependence by the NMDA receptor antagonist MK-801. Science.

[CR48] Tsuda M, Suzuki T, Misawa M (1997). Recovery of decreased seizure threshold for pentylenetetrazole during diazepam withdrawal by NMDA receptor antagonists. Eur J Pharmacol.

[CR49] Tsuda M, Suzuki T, Misawa M (1997). Role of the NMDA receptor complex in DMCM-induced seizure in mice. Neuroreport.

[CR50] Tsuda M, Chiba Y, Suzuki T, Misawa M (1998). Upregulation of NMDA receptor subunit proteins in the cerebral cortex during diazepam withdrawal. Eur J Pharmacol.

[CR51] Tsuda M, Suzuki T, Misawa M (1998). NMDA receptor antagonists potently suppress the spontaneous withdrawal signs induced by discontinuation of long-term diazepam treatment in Fischer 344 rats. Brain Res.

[CR52] Tsuda M, Shimizu N, Yajima Y, Suzuki T, Misawa M (1998). Hypersusceptibility to DMCM-induced seizures during diazepam withdrawal in mice: evidence for upregulation of NMDA receptors. Naunyn Schmiedeberg’s Arch Pharmacol.

[CR53] Tsuda M, Shimizu N, Yajima Y, Suzuki T, Misawa M (1998). Role of nitric oxide in the hypersusceptibility to pentylenetetrazole-induced seizure in diazepam-withdrawn mice. Eur J Pharmacol.

[CR54] Uzbay IT, Erden BF, Tapanyigit EE, Kayaalp SO (1997). Nitric oxide synthase inhibition attenuates signs of ethanol withdrawal in rats. Life Sci.

[CR55] Vaupel DB, Kimes AS, London ED (1997). Further in vivo studies on attenuating morphine withdrawal: isoform-selective nitric oxide synthase inhibitors differ in efficacy. Eur J Pharmacol.

[CR56] Ward BO, Stephens DN (1998). Sensitisation of withdrawal signs following repeated withdrawal from a benzodiazepine: differences between measures of anxiety and seizure sensitivity. Psychopharmacology.

[CR57] Wazlawik E, Morato GS (2002). Effects of intracerebroventricular administration of 7-nitroindazole on tolerance to ethanol. Brain Res Bull.

